# ACCEPTABILITY OF THE RESOURCE CONNECTION FOR RURAL CAREGIVERS OF PEOPLE LIVING WITH DEMENTIA

**DOI:** 10.1093/geroni/igae098.2263

**Published:** 2024-12-31

**Authors:** Sydney Hoel, Lillian White, Matthew Zuraw, Jordan Hill, Christian Elliot, Andrew Pickett, Nicole Werner

**Affiliations:** Indiana University, Bloomington, Bloomington, Indiana, United States; Indiana University Bloomington, Bloomington, Indiana, United States; CareVirtue, San Diego, California, United States; Indiana University Bloomington, Bloomington, Indiana, United States; CareVIrtue, San Diego, California, United States; Indiana University, Bloomington, Bloomington, Indiana, United States; Indiana University, Bloomington, Bloomington, Indiana, United States

## Abstract

Rural family caregivers of people living with Alzheimer’s disease and related dementias (ADRD) experience higher caregiver burden and lower social supports compared to urban ADRD caregivers. However, research regarding how to better connect rural ADRD caregivers to resources in the community is lacking. CareVirtue Resource Connection, a web-based platform designed with rural ADRD caregivers and rural community organizations, aims to provide rural ADRD caregivers with personalized and hyper-localized resources. We conducted a 30-day field test of Resource Connection with rural ADRD caregivers (N=15) followed by semi-structured interviews to explore participants’ perceptions of CareVirtue Resource Connection acceptability. The objective of this study was to identify barriers and facilitators to Resource Connection acceptability based on the post-use interviews. Interview transcripts were analyzed using deductive content analysis guided by the Technology Acceptance Model (i.e., ease of use and usefulness) using an iterative team-based coding process. ADRD caregivers were self-identified rural, aged Mean=70.1 years (59-81 years), 80.0% women (n=12), and 93.3% white (n=14). Facilitators to Resource Connection acceptability included having all resources provided in one space, resource recommendations based on need and location, notifications about new resources, connecting with other caregivers, emotional and mental health benefits, and the ability to share resource information with others. Acceptability barriers included limited time to connect with provided resources, mismatch of resources to ADRD stage, and a lack of local resources. Results suggest key areas for improving the design that will inform intervention refinement prior to large-scale effectiveness testing.

